# “Forced” Education

**Published:** 1899-11

**Authors:** 


### "Forced" Education.

The damage to the nervous organization of the child, and the generally disastrous effects of forcing in the school curriculum, have been often remarked, but the evil still continues. The *N. Y. Medical Journal* (Sept. 16th) notices with approval an address made before a provincial Canadian medical society last summer, in which this subject was taken up. The special point made was that the course of study should be carefully adapted to the average, or, rather, the under-average child, and this both as regards mental and physical development. "Rudimentary education is all that the community should set peremptorily before the mass of children; those who have the capacity for more advanced education are few in number, and their aspirations may well be met by a few high schools." These are truths, we might better say truisms, but our educational authorities do not always seem to regard them, and their repetition can not well be too emphatic. With all our boasted intelligence, we might as well recognize the fact that mediocrity is the rule and talent and genius are the exception.—*The Journal.*

